# Sodium hyaluronate and chondroitin sulfate replenishment therapy can improve nocturia in men with post-radiation cystitis: results of a prospective pilot study

**DOI:** 10.1186/s12894-015-0046-1

**Published:** 2015-07-07

**Authors:** Mauro Gacci, Omar Saleh, Claudia Giannessi, Beatrice Detti, Lorenzo Livi, Eleonora Monteleone Pasquetti, Tatiana Masoni, Enrico Finazzi Agro, Vincenzo Li Marzi, Andrea Minervini, Marco Carini, Stavros Gravas, Matthias Oelke, Sergio Serni

**Affiliations:** Department of Urology, University of Florence, Careggi Hospital, Largo Brambilla 3, Urologic Clinic San Luca, Florence, 50100 Italy; Department of Radiation Therapy, University of Florence, Careggi Hospital, Largo Brambilla 3, Florence, Italy; Department of Urology, Tor Vergata University, Via di Tor Vergata, Rome, Italy; Department of Urology, University Hospital of Larissa, Larissa, Greece; Department of Urology, Hannover Medical School, Hannover, Germany

**Keywords:** Prostate cancer, Acute radiation syndrome, Cystitis, Nocturia, Hyaluronic acid, Chondroitin sulfate

## Abstract

**Background:**

Radiotherapy is one of the treatment options for prostate cancer (PCa) but up to 25 % of men report about severe nocturia (nocturnal voiding). The combination of hyaluronic acid (HA) and chondroitin sulfate (CS) resembles glycosaminoglycan (GAG) replenishment therapy. The aim of our study was to evaluate the impact of HA and CS on nocturia, in men with nocturia after PCa radiotherapy.

**Methods:**

Twenty-three consecutive patients with symptomatic cystitis after external radiotherapy for PCa were enrolled. Patients underwent bladder instillation therapy with HA and CS weekly for the first month and, afterwards, on week 6, 8 and 12. Nocturnal voiding frequency was assessed by item 3 (Q3) of the Interstitial Cystitis Symptoms Index (ICSI) and item 2 (Q2) of the Interstitial Cystitis Problem Index (ICPI). Data were analyzed with paired-samples T-test and adjusted for age.

**Results:**

Eighteen patients (78 %) reported about nocturia. Pre- and post-treatment ICSI-Q3 was 2.13 ± 0.28 and 1.61 ± 0.21 (−24.4 %, p = 0.001). With logistic regression analysis, both age and baseline ICSI-Q3 had a significant impact on nocturnal voiding frequency (r = 0.293, p = 0.011 and r = 0.970, *p* < 0.001). Pre- and post-treatment ICPI-Q2 was 1.87 ± 0.26 and 1.30 ± 0.25 (−30.5 %, p = 0.016); logistic regression analysis was without significant findings.

**Conclusion:**

Bladder instillation treatment with a combination of HA and CS was effective in reducing nocturnal voiding frequency in men with post-radiation bladder pain for PCa. Randomized, controlled trials with sham treatment are needed to confirm our result.

## Background

According to international prostate cancer (PCa) guidelines, external beam radiotherapy (EBRT) can be applied as primary treatment and as an alternative to radical prostatectomy in patients with low to medium risk localized PCa (Gleason score ≤7 or cT ≤ 2c or PSA ≤ 20 ng/ml) [1, 2], or can be used after surgery as adjuvant or salvage treatment, in case of biochemical relapse [3].

New PCa treatment modalities, such as intensity modulated radiation therapy (IMRT), achieve to focus the maximum administered dose more precisely on the prostate, thereby avoiding radiation damage on adjacent organs such as the bladder. Nevertheless, even with IMRT, up to 50 % of patients treated with doses >70 Gy experience lower urinary tract symptoms (LUTS), especially during the early treatment period (acute radiation toxicity). Of these patients, up to 25 % report nocturnal voiding) during or immediately after radiation therapy [4, 5]. Patients with nocturia experienced a remarkable worsening of their quality of life. Higher the nocturnal voiding frequency was, the more deteriorated the HRQoL became. The reason can be attributed to the impaired sleep caused by nocturia. Sleep disruption, in particular during the first 4 hours of slow wave sleep (SWS) period, has been implicated as a likely mechanism underlying the subjective complaints, daytime tiredness, and depressive symptoms [6, 7]. It has been hypothesized that post-radiation LUTS, including nocturnal voiding frequency (nocturia), could be caused by the damage, disruption and, consequently, discontinuation of the glycosaminoglycan (GAG) layer of the bladder mucosa [8]. Therefore, GAG replenishment therapy by instillation of hyaluronic acid (HA) with or without chondroitin sulfate (CS) has been suggested as a viable treatment option to treat post-radiation LUTS [9]. Up until now, only few clinical trials have been published which investigated GAG replenishment treatment with HA and CS combination to treat bladder pain or post-radiation LUTS [10] and no study has ever investigated nocturia/nocturnal voiding frequency in post-radiation PCa patients. Therefore, the aim of this pilot study was to evaluate the efficacy and safety of intravesical GAG replenishment treatment with HA and CS combination in men with nocturia in men after radiation therapy for prostate cancer.

## Methods

### Patient population and study design

Male patients with urinary symptoms due to post radiation cystitis and negative urine cultures after external beam radiotherapy for PCa were enrolled between May 2012 and October 2013. Men with a history of previous bladder catheterizations for acute urinary retention, urinary tract infections or bladder stones or a known malignant disease besides PCa were excluded from this study. Age and comorbidities, Gleason score and serum PSA concentration as well as radiation dose and toxicity were recorded for all patients.

Men were treated with bladder instillations containing HA and CS combination (HA 1.6 % 800 mg/50 ml and CS 2 % 1000 mg/50 ml) according only to the indication and schedule reported in the package leaflet of the manufacturer (Ialuril® Ibsa Pambio-Noranco, Switzerland). Pre- and post-treatment LUTS, including nocturnal voiding frequency, were assessed by validated questionnaires. The study protocol was reviewed and approved by the local ethic committee in Florence, Italy (A.O.U.C. Careggi ethic committee). The study did not require any deviation of the current clinical practice standards and was conducted in accordance to the principles of research, as reported in the Declaration of Helsinki or in Good Clinical Practice standards for PCa. All patients received all information about the study design and they signed informed consent. No children were included in the study.

### Radiotherapy treatment

The planning computed tomography scan was performed with 3-mm slices, with the patient in the supine position and using a leg immobilization system (Combifix-Sinmed, Civco, Kalona, IA, USA). The total dose applied was 66–70 Gy (2 Gy/fraction, five fractions weekly,). The clinical target volume (CTV) was limited to the prostatic bed and periprostatic tissue, ensuring adequate coverage of the vesico-urethral anastomosis. The planning treatment volume (PTV) included the clinical target volume, plus a 10-mm margin in all directions. For postoperative treatment, 66–70 Gy in 33–35 fractions were delivered with a tridimensional conformal technique (3DCRT).

### Hyaluronic acid and chondroitin sulfate administration and assessment of cystitis and nocturia

Three months after radiation therapy, patients with urinary symptoms due to radiation cystitis underwent intravesical administration of HA and CS weekly for the first month, and on week 6, 8 and 12 as reported in the package leaflet of the manufacturer. HA and CS was diluted in 50 ml physiologic NaCl solution, placed inside the bladder with sterile catheters (14 F) and retained for at least 1 h.

At baseline and 2 weeks after the end of treatment (week 12 + 2 = week 14), all patients were asked to complete the Interstitial Cystitis Symptom Index and Problem Index (ICSI/ICPI), initially proposed in 1997 as outcome measures in bladder pain syndrome/interstitial cystitis (BPS/IC) and currently recognized as one of the most accurate tools to identify the most relevant voiding and pain symptoms due to bladder pain [11]. In particular, the *symptom* nocturnal voiding was measured with the question 3 (Q3) of ICSI asking: *How often did you most typically get up at night to urinate?* Answers range from 0 (“not at all”) to 5 (“5 or more times per night”). The *problem* associated with nocturia was measured with question 2 (Q2) of ICPI asking: *How much has getting up at night to urinate been a problem for you?* Answers range from 0 (“no problem”) to 4 (“big problem”).

### Statistical analyses

Correlation between symptoms and problems detected at the ICSI and ICPI total score and for the specific ICSI-Q3 and ICPI-Q2 at baseline and at the end of the study was performed with the Spearman correlation coefficient; significant data were included in an age adjusted model. Mean changes between baseline and the end of study (week 14) were assessed by a paired sample t test, for ICSI and ICPI total score and for the specific ICSI-Q3 and ICPI-Q2. A p value of 0.05 or less was considered statistically significant. All statistical analyses were done with SPSS-20®.

## Results and discussion

We included 23 consecutive patients with symptomatic cystitis and negative urine cultures after RT. Five patients (21.7 %) did not have nocturnal voiding (even if they were affected by other symptoms of cystitis), while the remaining 18 (78.3 %) presented with nocturnal voiding frequency (nocturia): 7 patients (38.9 %) 2 times per night, 10 patients (35.7 %) 3 times per night and 1 patient (3.4 %) 5 times per night. Median age of men with nocturnal voiding was 70.5 ± 5.9 years. Eleven were affected by high risk PCA (61 %), 6 by intermediate risk (33 %) and 1 by low risk (6 %), according to PCa risk classification [12]. Median dose that patients received was 67.9 ± 2 Gy and all patients reported at least grade 2 of toxicity according RTOG adverse event reporting [13].

**Fig. 1:**
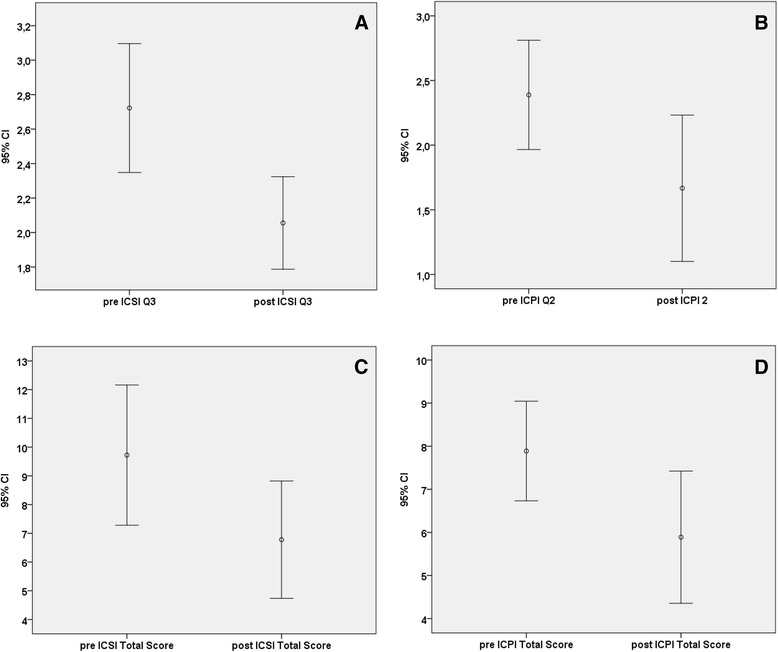
Pre and post treatment mean value of ICSI-Q3 (**a**), ICPI-Q2 (**b**), ICSI Total score (**c**) and ICPI Total score (**d**). Bars represent 95 % confidence intervals. The relative adjusted and level of significance (p) are reported in the text

Men with nocturnal voiding presented a statistically significant recovery of this symptom, after treatment with HA and CS (Table 1). In particular, there was a significant reduction of ICSI/ICPI total score and ICSI-Q3 and ICPI-Q2 between pre-treatment baseline and post-treatment evaluation (Fig. 1 and Table 2). Specifically regarding symptoms (ICSI Q3), 10 patients (56 %) showed improvement in the number of nocturnal voids, 8 patients (44 %) had no change and no patient reported worsening. Regarding bother (ICPI Q2), 10 patients (56 %) showed improvement, 7 patients (39 %) had no change, and only 1 patient reported worsening. At the end of bladder instillation treatment, men without nocturia at baseline continued to be without nocturia: therefore, these 5 men were excluded by further statistical analyses (Table 3).

**Table 1:** Number of nocturnal voids at baseline and week 14

ICSI Q3 score				
Baseline frequency	N	End point (week 14)		
		1	2	3
2	7	1	6	-
3	10	1	7	2
4	0	-	-	-
≥5	1	-	-	1

**Table 2:** Pre and postoperative mean score and standard deviation of ICSI / ICPI (total score-Q3-Q2)

		Pre treatment	Post treatment	P value(pairT-test)
ICSI	Q3	2.72 ± 0.75	2.06 ± 0.53	0.001
	Total score	9.72 ± 4.91	6.78 ± 4.11	0.004
ICPI	Q2	2.39 ± 0.85	1.67 ± 1.14	0.015
	Total score	7.89 ± 2.32	5.89 ± 3.08	0.006

.

**Table 3:** Distribution of patients with improvement, no change or worsening at end of treatment, by baseline score

ICSI Q3				
Baseline frequency	N	Improvement N (%)	No change N (%)	Worsening N (%)
2	7	1 (4 %)	6 (86 %)	0 (0 %)
3	10	8 (80 %)	2 (20 %)	0 (0 %)
4	0	0 (0 %)	0 (0 %)	0 (0 %)
≥5	1	1 (100 %)	0 (0 %)	0 (0 %)
All	18	10 (56 %)	8 (44 %)	0 (0 %)
ICPI Q2				
Baseline frequency	N	Improvement N (%)	No change N (%)	Worsening N (%)
0	1	0 (0 %)	0 (0 %)	1 (100 %)
1	1	1 (100 %)	0 (0 %)	0 (0 %)
2	6	3 (50 %)	3 (50 %)	0 (0 %)
3	10	6 (60 %)	4 (40 %)	0 (0 %)
4	0	0 (0 %)	0 (0 %)	0 (0 %)
All	18	10 (56 %)	7 ( 39 %)	1 ( 5 %)

We did not find any significant correlation between “nocturia symptom” and “nocturia bother” (ICSI-Q3 vs ICPI-Q2: baseline: r = −0.110, p = 0.664; end of study: r = 0.549, p = 0.151) before and after instillation therapy. Moreover, comorbidities, tumor characteristics, including PSA and Gleason Score, dose radiation and toxicity were not correlated with ICSI-Q3 or ICPI-Q2 (data not shown).

At baseline (after radiation therapy) ICSI-Q3 scores were correlated with ICSI total scores (r = 0.649, p = 0.004), and ICPI-Q2 scores were correlated with ICPI total scores (r = 0.472, p = 0.048). At the end of study (after instillation), ICSI-Q3 scores were correlated with ICSI total score (r = 0.466, p = 0.051), and ICPI-Q2 scores were correlated with ICPI total scores (r = 0.808, *p* < 0.001). Post-treatment ICSI-Q3 scores correlated with pre-treatment ICSI-Q3 scores (r = 0.402, p = 0.048), while post treatment ICPI-Q2 scores correlated with pre-treatment ICPI-Q2 scores (r = 0.536, p = 0.022).

After age adjustement, both age and baseline ICSI-Q3 correlated with post-treatment ICSI-Q3 (r = 0.293, p = 0.011 and r = 0.970, p < 0.001, respectively), while no significant data were seen in multivariate analyses for post-treatment ICPI-Q2.

Genitourinary toxicities during or after radiotherapy are a common finding for men treated for prostate cancer. In particular, up to 25 % of these patients report about nocturia as a bothersome treatment-related adverse event which can have a substantial impact on patients’ quality of life [4, 14, 15]. Several factors, including comorbidities (metabolic syndrome, hearth failure, chronic renal failure), existing lower urinary tract symptoms (related to benign prostatic enlargement), or urinary tract infections can be predictive for post-radiation bladder toxicity [15]. However, the physical injury of the bladder urothelium is most likely the main pathogenetic factor of post-radiation bladder symptoms, including nocturia. The damage can be related to the volume and dose of radiation. 65 Gy has been estimated as the maximum tolerated dose for whole bladder field irradiation in order to maintain severe urinary toxicity below 5 %, while with 80 Gy the occurrence of bladder injury can be observed in up to 50 % of patients [16]. For the treatment of PCa, the bladder is only partly irradiated. However, when bladder, bladder neck and urethra receive radiation doses >70 Gy, bothersome urinary symptoms, including nocturia, are commonly observed [17].

Post-radiation injury of bladder urothelium can be caused by specific damage of the GAG layer. GAGs are a class of polysaccharides, of which HA and CS are key components. When damaging this layer, penetration of potassium into the bladder wall (interstitium) can cause activation of C-fibers which promote smooth muscle contraction, neurogenic inflammation, and hypersensitivity [18, 19]. Therefore, the aim of the treatment of radiation cystitis includes replanishment of the GAG layer of the bladder and reduction of mast cell mediated inflammatory cascades. Many other treatments have been proposed including hyperbaric oxygen therapy (HBOT) [20] since it is postulated that HBOT may result in both healing of tissues and the prevention of problems following radiotherapy; intravesical instillation with dimethyl sulfoxide [21]; or several antimuscarinic (anticholinergics) drugs such as tolterodine [22], α-adrenoceptor antagonists (α-blockers) and non-steroidal anti-inflammatory drugs. All treatments showed, however, poor clinical responses [23].

Recent papers have suggested that CS could promote regeneration of the GAG layer on the bladder urothelium and HA could have an inhibitory effect on mast cells of the bladder. In our population of men with post-radiation bladder symptoms, the severity of nocturia did not correlate with the severity of bother due to nocturia suggesting that immediately after radiation patients are more concerned by other symptoms, including bladder pain. Moreover, in our patients the presence of comorbidities, tumor characteristics (PSA and Gleason score), dose radiation and toxicity had a negligible influence on nocturnal voiding and nocturia-related bother.

The association between nocturnal voiding (ICSI-Q3 and ICPI-Q2) and the whole pattern of bladder pain symptoms and bother (ICSI/ICPI total scores) demonstrated that nocturnal voiding frequency (nocturia) is only one of the symptoms due to the injury of the bladder and restoration of the bladder wall after treatment allow to relief not only on nocturnal voiding, but also other post-radiation bladder symptoms. These data are confirmed by our age adjusted analysis, demonstrating that elderly men with the worst nocturnal voiding symptoms are those with poorer outcomes after instillation therapy. In these patients, the bladder damage seems to be only partially restored by replenishment therapy, resulting in persistent nocturia. Overall, treatment with HA and CS administration was well tolerated, with no serious adverse event or early treatment discontinuations. In particular, no patients, including those without nocturnal voids, reported any worsening of their local and systemic symptoms, including the occurrence of fever or hematuria.

The main limits of our study are the small sample size, the short follow up time and the lack of a placebo arm. Our small sample size could be considered satisfactory for a “pilot study design”; however, to better understand the timing of the response to treatment with Ialuril, and to measure the difference between spontaneous recovery of urinary function vs. treatment related clinical effect, a randomized, placebo controlled trial, with several scheduled symptoms assessments and adequate follow up time is needed. The strengths are the uniformity for radiation treatment, the use of validated questionnaire and the adjustment for confounding factors.

## Conclusions

In conclusion, our pilot study demonstrated that bladder instillation with HA and CS is a safe treatment in post radiation bladder cystitis. Patients after Ialuril ® instillation reported a statistically significant reduction in symptom/bother nocturnal voiding frequency, even if the overall improvement due to the medication vs. spontaneous recovery must be confirmed by placebo controlled trials, including further assessment tools such as cystoscopy and histological evaluation.

## Abbreviation

(PCa): Prostate cancer
(HA): Hyaluronic acid
(CS): Chondroitin sulfate
(GAG): Glycosaminoglycan
(ICSI): Interstitial Cystitis Symptoms Index
(ICPI): Interstitial Cystitis Problem Index
(EBRT): External beam radiotherapy
(IMRT): Modulated radiation therapy
(LUTS): Lower urinary tract symptoms
(CTV): Clinical target volume
(PTV): Planning treatment volume
(3DCRT): Tridimensional conformal technique
(BPS/IC): Bladder pain syndrome/interstitial cystitis
(HBOT): Hyperbaric oxygen therapy
